# The Commonality of Loss Aversion across Procedures and Stimuli

**DOI:** 10.1371/journal.pone.0135216

**Published:** 2015-09-22

**Authors:** Sang Lee, Myung J. Lee, Byoung W. Kim, Jodi M. Gilman, John K. Kuster, Anne J. Blood, Camelia M. Kuhnen, Hans C. Breiter

**Affiliations:** 1 Warren Wright Adolescent Center, Northwestern University Feinberg School of Medicine, Chicago, IL, United States of America; 2 Laboratory of Neuroimaging and Genetics, Department of Psychiatry, Massachusetts General Hospital (MGH) and Harvard Medical School (HMS), Boston, MA, United States of America; 3 Mood and Motor Control Laboratory, MGH and HMS, Boston, MA, United States of America; 4 Kenan-Flagler Business School, University of North Carolina, Chapel Hill, NC, United States of America; 5 Massachusetts General Hospital and Northwestern University Phenotype Genotype Project in Addiction and Mood Disorders; University of Sheffield, UNITED KINGDOM

## Abstract

Individuals tend to give losses approximately 2-fold the weight that they give gains. Such approximations of loss aversion (LA) are almost always measured in the stimulus domain of money, rather than objects or pictures. Recent work on preference-based decision-making with a schedule-less keypress task (relative preference theory, RPT) has provided a mathematical formulation for LA similar to that in prospect theory (PT), but makes no parametric assumptions in the computation of LA, uses a variable tied to communication theory (i.e., the Shannon entropy or information), and works readily with non-monetary stimuli. We evaluated if these distinct frameworks described similar LA in healthy subjects, and found that LA during the anticipation phase of the PT-based task correlated significantly with LA related to the RPT-based task. Given the ease with which non-monetary stimuli can be used on the Internet, or in animal studies, these findings open an extensive range of applications for the study of loss aversion. Furthermore, the emergence of methodology that can be used to measure preference for both social stimuli and money brings a common framework to the evaluation of preference in both social psychology and behavioral economics.

## Introduction

Decision-making depends on the underlying preferences people have to approach some items/events and avoid others, an idea formalized under the expected utility hypothesis [1]. An important assumption of the expected utility hypothesis is that people maximize their expected utility during decision-making, but empirical data does not always support this [2]. One potential explanation for these observations has been proposed as the concept of “loss aversion” (LA), whereby people consider outcomes as gains or losses relative to a reference point, and appear more sensitive to losses than to similar size gains [3]. Formalized by prospect theory (PT) in the context of monetary gambles [3], LA has been quantified across multiple studies [4–6], and can explain a broad array of data [7–9] and human experiments [10–11]. This success has occurred despite the observation that LA measures may differ depending on the phase of cognitive processing tested–whether a person is (i) making a decision, (ii) anticipating an outcome of a decision, or (iii) experiencing a decision outcome [12]. Furthermore, two general definitions of LA are being used by research teams, a “global” and a “local” definition [13–16], which inject variance into comparisons.

Global definitions of LA [3,14] are measured over the entire value function, and do not define LA separate from utility curvature (i.e., risk aversion)[17,18]. In contrast, local definitions of LA measure LA proximate to the inflection point in the value/utility function (e.g., often the origin of the graph)[15,16], and can separate LA from other components of risk attitude, including utility curvature and probability weighting. This global vs. local distinction is important [13], given the location where LA is measured on the value/utility curve affects results (i.e., the value function is steeper near the origin)[19].

Under the aegis of PT, LA has been defined as (i) the slope of the negative value/utility function (s-) compared to (ii) the slope of the positive value/utility function (s+), approximating the absolute value of s-/s+ (i.e., |s-/s+|)[3,16,20]. Across studies, people give losses from 1.74 to 4.80 times the weight that they give gains [13]. These estimates are almost always measured in the stimulus domain of money (i.e., not objects or pictures), which limits their generalizability to animal studies, to populations with different financial assets, or to the web. Recent work on preference-based decision-making with a schedule-less keypress task has provided a formulation for |s-/s+| that appears mathematically related to LA in PT, makes no parametric assumptions in the computation of |s-/s+|, and works readily with non-monetary stimuli. Referred to as relative preference theory (RPT)[21], two of the variables in RPT describe a value function with a power law formulation strikingly similar to that in PT. One of these RPT variables reflects the mean level of keypressing (K) while the other (H) reflects the amount of uncertainty associated with a choice (i.e., information) computed using Shannon’s entropy equation from Communication Theory [22]. RPT encodes salient features of alliesthesia or hedonic deficit theory [23,24], variance-mean theory (portfolio theory)[25], and matching (stimulus-response learning)[26]. RPT variables have been associated with neural activation in regions that others have associated with measures of LA [27–30], such as the amygdala and nucleus accumbens [31,32]. RPT variables have also been used in imaging genetics as the behavioral metric associated with neural activation in amygdala, nucleus accumbens, and other reward/aversion regions, which in turn act as intermediary phenotypes for association to genetic polymorphisms for CREB1 [33] and BDNF [32]. RPT-based estimates of |s-/s+| make no assumptions regarding convexity or concavity of the negative or positive value/utility curves [21], and show an overweighting of negative versus positive valuation, which will also be referred to as LA.

In this study, we sought to evaluate if distinct variables involved with the definition of LA or distinct frameworks for LA described similar parameter estimates, even if these frameworks differed with regard to (A) the stimulus used (money vs. non-monetary stimuli), (B) measure of behavior (ratings vs. keypress), (C) mode of LA measured (global vs. local loss-aversion), or (D) variables used to calibrate value [i.e., (i) gains/losses across group vs. individual utility in PT, and (ii) the mean keypress for stimuli (i.e., wanting) vs. the pattern of responses in RPT]. To test each component of this question, we had the same subjects participate in a PT-based game of chance, using monetary reward, along with a keypress paradigm [31] involving a stimulus set depicting categorical facial expressions [34], which allowed an RPT-based mapping of relative preferences toward social stimuli across subjects [21]. By testing preference to both money and social stimuli, we were able to evaluate whether a common framework could be used for measures of preference in both a behavioral economics (i.e., neuroeconomic) and a social psychology context.

## Methods

### Subjects

Twenty-three healthy control subjects completed both the PT-based monetary task and the RPT-based keypress task. One subject of these twenty-three was excluded based on their behavioral data (see analysis below). The final cohort of 22 subjects was characterized as follows: age = 31.18 ± 10.78 years, gender = 13 males, right handed = 19, race = 18 Caucasian, 2 African American, 2 Asians, education = 15.00 ± 1.98 years. Women were scanned during their mid-follicular phase based upon self-reported menstrual history, with confirmation at the time of testing based on hormonal testing with a urine assay.

All subjects were recruited by advertisements and underwent a clinical interview with a doctoral-level clinician, which included the Structured Clinical Interview for Diagnosis–Axis I [35], along with medical evaluation which included a physical exam, review of systems, and blood chemistry by a board certified physician. Race was determined by individual self-identification using a standardized form [36], and handedness via the Edinburgh Handedness Inventory [37]. Participating subjects were age 18–55, without any current or lifetime DSM-IV Axis I disorder or major medical illness known to influence brain structure or function, including neurologic disease, HIV, and hepatitis C. Subjects were scanned at normal or corrected normal vision.

### Ethics Statement

**Fig 1:**
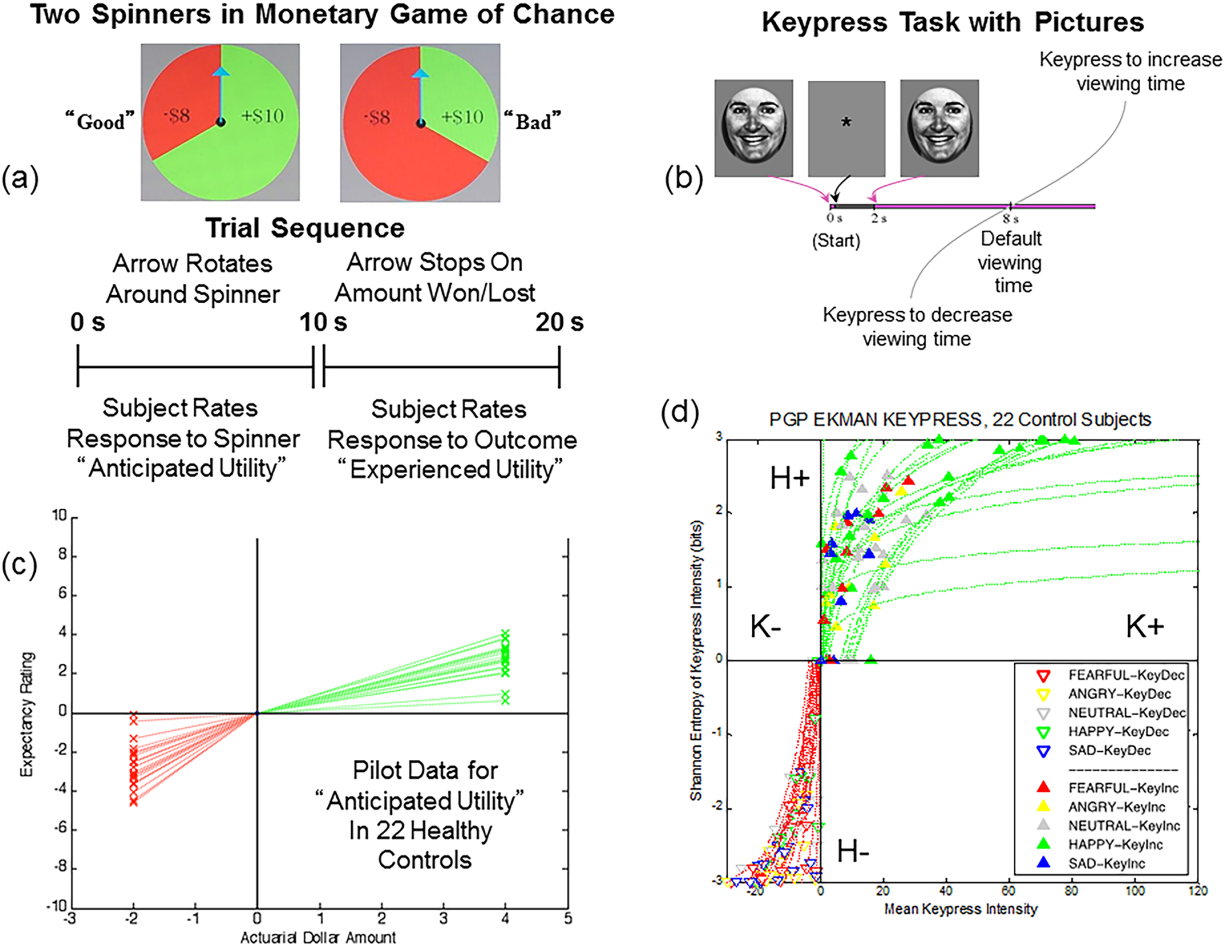
Experimental Procedures and Resulting Value Functions. **(a)** The PT-based experiment used two “gambles”, schematized by two spinners. One spinner showed two-thirds of its area as gains (+$10) and one-third as losses (-$8), leading to an expected outcome (i.e., referred to as actuarial outcome in Breiter et al. [38]) of +$4. The second spinner showed one-thirds of its area as gains (+$10) and two-thirds as losses (-$8), leading to an expected outcome of-$2. Each trial lasted 20 seconds, with 10s focused on the arrow spinning (anticipation phase) and 10s focused on the arrow stopping, and the win/loss flickering (outcome phase). Order of presentation between the PT-based experiment and RPT-based experiment was counterbalanced across subjects. **(b)** The RPT-based experiment used a keypress procedure [21]: a picture would appear for 200ms, then be replaced by a fixation point for 1800ms. After 2000ms, the face would reappear, and if subjects did nothing, the face would stay up another 6000ms (e.g., default condition). Subjects could increase viewing time via a scalloping resistive function, getting close to maximum 1400ms. Alternatively, they could decrease viewing time with the same resistive function close to a minimum of 2000ms. The scalloping resistive function incrementally reduced the viewing time alteration achieved by each keypress, so subjects needed to exert effort to increase or reduce viewing times. Its mathematical formulation can be found in Kim et al. [21], along with multiple control analyses about its impact on subject behavior. **(c)** The value function for the PT-based experiment mapped subjective ratings made during the anticipation phase of the experiment on the y-axis, and the actuarial amount of the spinner on the x-axis. For the outcome phase of this experiment, the value function mapped the subjective ratings made when the arrow stopped spinning against the gain or loss. **(d)** The RPT-based graph showed the mean intensity of keypressing to increase viewtime (K_+_) or decrease viewtime (K_-_) calibrated against the Shannon entropy of keypress patterns to increase (H_+_) or decrease (H_-_) viewtime. Solid and empty triangles stand for individual data points for the five categories of facial expressions.

All subjects signed written informed consent prior to participation, for this study approved by the Institutional Review Board of Massachusetts General Hospital (i.e., Partners Human Research Committee, Partners Healthcare), and all experiments were conducted in accordance with the principles of the Declaration of Helsinki. The individual shown in Fig 1 of this manuscript was part of the Ekman and Friesen stimulus set [34], a validated and disseminated stimulus set. As part of this published stimulus set, this individual has given consent to publish their image.

### Experimental Paradigms and Data Acquisition

#### Monetary Game of Chance (PT framework)

For a task in the framework of PT, we used a monetary game of chance task adapted from Breiter et al. [38], with controlled expectancy and outcomes that followed PT. Two spinners with different weightings of gain (+$10) and loss (-$8) were used, with expected outcomes of-$2 (Bad Spinner), and +$4 (Good Spinner) (see Fig 1A legend). Behaviorally, subjects made ratings of their emotional responses to the spinners with a potentiometer while an arrow was rotating around the spinner, reflecting their anticipated utility. They also rated their emotional response during the outcome phase when the arrow stopped rotating, and the sector on which it landed flickered, indicating the subject had won or lost that amount of money (reflecting outcome utility). Subjects rated their experiences of these two phases using a keypress to move a cursor across the screen for numeric values from -10 to +10. Expectancy and outcome components of the task each lasted 10 seconds; this time window gave subjects enough time to converge on a rating. Expectancy and outcome combinations were counterbalanced across trials.

#### Keypress Task (RPT framework)

For the non-monetary task outside the framework of PT, we used a validated keypress procedure derived from the operant conditioning literature [31–33,21]. The keypress procedure was implemented with MatLab software on a PC (i.e., a personal computer). As performed in other published studies, this task captured the reward valuation attributed to each observed face, and quantified positive and negative preferences involving (i) decision-making regarding the valence of behavior, and (ii) judgments that determine the magnitude of approach and avoidance [39,33,32,21]. The objective was to determine how much effort each subject was willing to trade for viewing each facial expression compared to a default viewing time (Fig 1B). Subjects were told that they would be exposed to a series of pictures that would change every eight seconds (the default valuation of 6 seconds + 2 second decision block; Fig 1B) if they pressed no keys. If they wanted a picture to disappear faster, they could alternate pressing one set of keys (#3 and #4 on the button box), whereas if they wanted a picture to stay longer on the screen, they could alternate pressing another set of keys (#1 and #2 on the button box). Subjects had a choice to do nothing (default condition), increase viewing time, decrease viewing time, or a combination of the two responses (Fig 1). A “slider” was displayed to the left of each picture to indicate total viewing time. Subjects were informed that the task would last approximately 20 minutes, and that this length was independent of their behavior, as was their overall payment. The dependent measure of interest was the amount of work, in number of keypresses, which subjects traded for face viewtime. Keypress results could also be expressed as total viewtime relative to the default baseline

In this study, the pictures were emotional facial expressions for angry, fearful, sad, neutral, and happy faces, from the Ekman and Friesen set [34], normalized following procedures described in Breiter et al. [40].

### Data Analysis

#### Monetary Game of Chance (PT framework)

For the monetary game of chance, ratings made during the expectancy phase of the task were graphed against the expected outcomes (actuarial values) for each spinner (Fig 1C). One subject of these twenty-three produced a positive rating for the “bad spinner” [see Experimental Paradigms, b. Spinner Task (PT framework)] which was the only positive rating made for this stimulus by anyone in the cohort. It was classified as an extreme outlier because the rating exceeded three times the interquatile range (IQR) above the third quartile (Q3). Before excluding this subject, the ratings for the “good spinner” were normally distributed (df = 23, p > .76) but the ratings for the “bad spinner” were not (df = 23, p < .0002), as assessed by Shapiro-Wilk's test. When this subject’s data was removed from the sample, the rating data of the cohort were normally distributed, as assessed by the same test (df = 22, p > .2), and the Grubb’s test did not detect any outliers ([41]; http://graphpad.com/quickcalcs/grubbs1/).

Ratings made during the outcome phase of each trial were graphed against the gain or loss in the spinner sector where the arrow landed. Following procedures for a global LA estimate [3,14], estimates were made for s- and s+, allowing computation of the absolute value for s-/s+ with the individual graphs in Fig 1D. LA estimates for data from the expectancy phase of the experiment were computed separately from LA estimates for data from the outcome phase.

#### Keypress Task (RPT framework)

**Fig 2:**
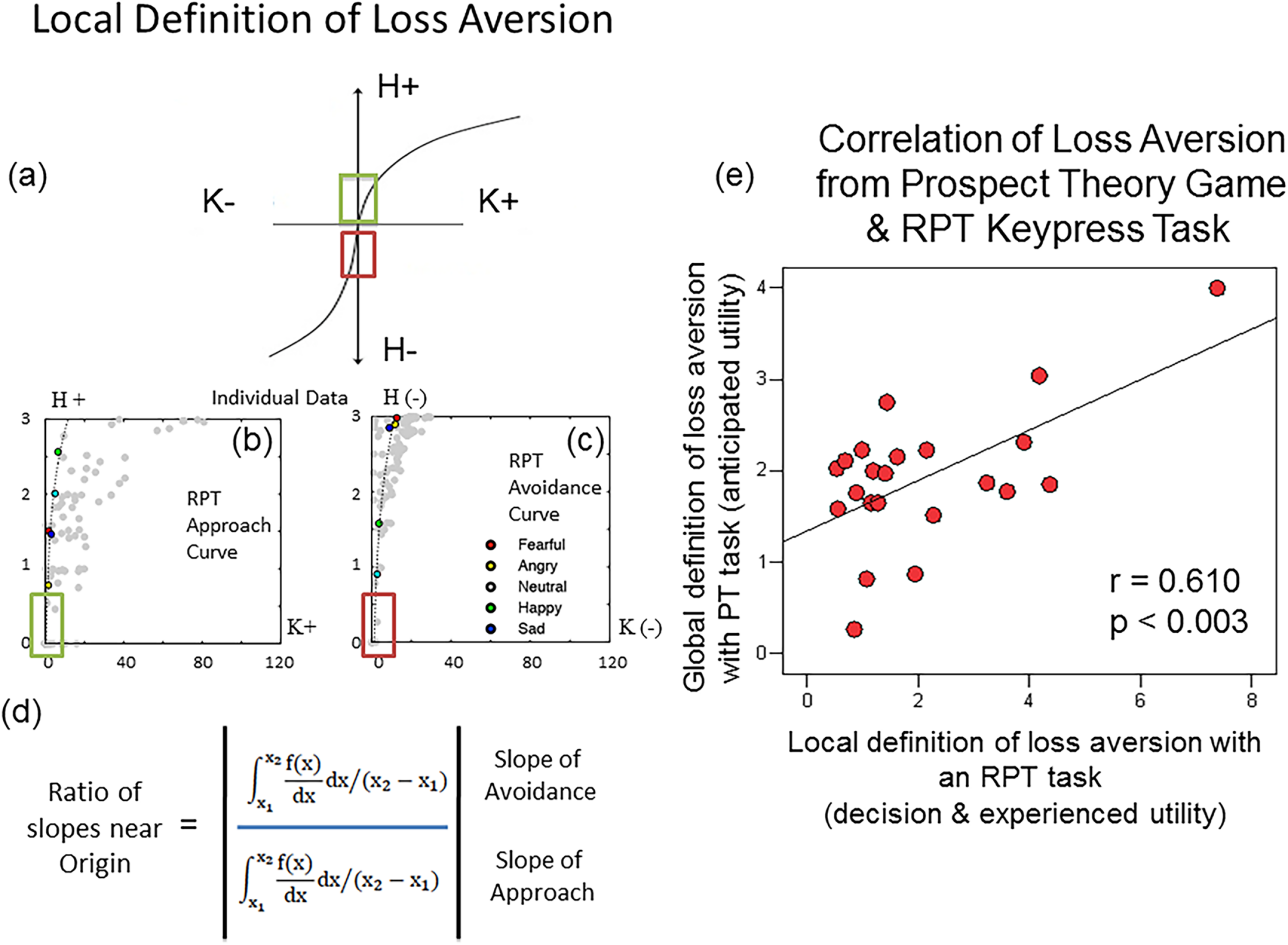
Definition of Loss Aversion (LA) and Correlation Between Measures. **(a)** The local definition of LA focuses on the slopes of the value function on the either side of an inflection point between approach and avoidance (s+ and s- respectively), or gains and losses. Measures of s+ and s- are collected close to the origin (see green and purple boxes), where the scale of value will minimally bias assessments of risk. The slopes **(b)** s+ and **(c)** s- are schematized for two representative curves from one individual. **(d)** LA is computed by the absolute value of the ratio of s- to s+, and is summed over the 10% of the graph on either side of the origin or inflection point. LA from this graph is quite similar to that reported by Kahneman and Tversky [5,42]. **(e)** Correlation of LA from the anticipatory phase of the PT-based task and from the RPT-based task, showing a significant effect after correction for multiple comparisons.

For the 22 subjects used for the PT task, raster plots of approach and avoidance responses to pictures (e.g., Fig 1B) were analyzed following procedures in Kim et al. [21], to produce the value functions shown in Fig 1D. These RPT-based value functions showed the mean intensity of keypressing to increase viewtime (K_+_) or decrease viewtime (K_-_) calibrated against the Shannon entropy of keypress patterns to increase (H_+_) or decrease (H_-_) viewtime. A local definition of LA was applied to these value functions [13] as schematized in Fig 2A. Specifically, s- and s+ were computed by the integral of the curve-fit slope over the 10% of the curve closest to the inflection point or origin (Fig 2B–2D). An absolute value of s-/s+ was then computed for each subject.

#### Between Task Assessments

We employed Pearson correlations (SPSS, version 13) for LA measures for the three possible pairings of the following three experimental conditions: (i) anticipation phase of the monetary task, (ii) outcome phase of the monetary task, and (iii) the keypress task (e.g., Fig 2E). A significant relationship between the two variables of each pair was required to meet a Bonferroni correction of p < 0.05/3 = 0.017 to correct for multiple comparisons.

## Results

Loss aversion (LA) for the anticipation phase of the PT-based monetary task involved a “global” measure which produced mean ± SE estimates for s+, s-, and the absolute value of s-/s+ (i.e., |s-/s+|) of 0.69 ± 0.05, 1.37 ± 0.12, and 1.93 ± 0.16, respectively. For the outcome phase of the PT-based monetary task, s+, s-, and the absolute value of s-/s+ (i.e., |s-/s+|) were 0.35 ± 0.03, 0.39 ± 0.04, and 1.11 ± 0.05, respectively.

LA for the RPT-based keypress task involved a “local” estimate, producing s+, s-, and an absolute values of s-/s+ (i.e., |s-/s+|) of 1.39 ± 0.53, 1.49 ± 0.51, and 2.12 ± 0.36, respectively.

The 95% confidence intervals for two of the three LA estimates, namely from the anticipation phase of the PT-based task, and from the RPT-based keypress task, overlapped the estimate of 2.25 published by Kahneman and Tversky [5,42], with the RPT-based keypress task producing an LA estimate very close to it. There was a strong positive correlation between anticipation LA (via the PT-based monetary task) and LA with the RPT-based keypress task (Fig 2E), *r*(20) = .610, p < .0003, and the location estimate for anticipation LA was within the 95% confidence interval for RPT-based LA.

In contrast, correlations between LA estimates for the anticipation and outcome phases of the PT-based monetary task were not significant (all p>0.05), and LA for the anticipation phase was almost double that of LA for the outcome phase, with the outcome phase showing almost no LA (i.e., a value close to 1). Similarly, the outcome LA estimate of the PT-based monetary task and that of the RPT-based keypress task were not significantly correlated. The correction for multiple comparisons was p < 0.05/3 = 0.017, for three comparisons.

All data from this experiment can be found in S1 Table.

## Discussion

These data provide an example in which a task based on PT, using monetary stimuli, and a task based on RPT, using non-monetary stimuli, produce similar loss aversion estimates. The correlation between the anticipation phase of the monetary game of chance and the keypress task occurred despite these tasks differing in four domains: (A) the stimulus used (money vs. non-monetary stimuli), (B) measure of behavior (ratings vs. keypress), (C) mode of LA measured (global vs. local loss-aversion), or (D) variables used to calibrate value. Although the keypress task does not contain overtly specified intervals regarding decision, anticipation, and outcome components of decision-making, it could be conceptualized as a task involving serial “micro-decisions” (i.e, keypress actions), followed by micro-intervals of viewing time (“outcomes”), with a relationship resembling an FR1 schedule in reinforcement learning, and each outcome interval determined by a scalloping resistive function [21]. Uncertainty is present in the form of viewing time remaining for each stimulus during its presentation, providing a potential rationale for why LA from the RPT task correlated with LA from the PT task anticipation phase, but not outcome phase. The absence of correlation between anticipation and outcome phases of the PT task is consistent with prior observations of differences in LA across phases of decision-making [12]. The similarity of LA estimates between the two tasks, despite significant differences in aforementioned domains (A)–(D), argues that LA may represent more of a general weighting between approach and avoidance decisions when uncertainty is present. Given RPT encodes critical features of reward/aversion processing from hedonic deficit theory [23,24], variance-mean theory (portfolio theory) [25], and matching [26], these findings identify a route by which LA can be evaluated for all of these reward/aversion frameworks. Specifically, LA can be computed in the context of the estimated utility approach (from prospect theory), and the other approaches to reward/aversion function. Furthermore, these findings demonstrate that individuals have similar measures of preference for both monetary and non-monetary stimuli, suggesting that this methodology may help align, in the context of reward evaluation, the fields of behavioral economics and social psychology.

## Supporting Information

S1 Table Rows 1–22 represent study subjects, and include data from both the Monetary Game of Chance (PT Framework) and the Keypress Task (RPT Framework). Columns under “Expectancy rating” refer to subject ratings to the “good disk” or “bad disk” during the Expectancy phase of the task. Columns under “Outcome rating” refer to ratings during the Outcome phase of the task, when a subject received a win with the good disk (goodD_win), loss with the good disk (goodD_lose), win with the bad disk (badD_win), or loss with the bad disk (badD_lose). Loss aversion metrics for the expectancy phase of the PT task included the slope of the negative curve (s-) and the slope of the positive curve (s+), along with absolute value of their ratio, which is the technical definition of loss aversion. Loss aversion metrics for the outcome phase of the PT task included the slope of the negative curve (s-) and the slope of the positive curve (s+), along with absolute value of their ratio, which is the technical definition of loss aversion. The RPT task produced a number of variables, including the mean keypresses to approach the picture viewed (K+), the mean keypresses to avoid the picture viewed (K-), the Shannon Entropy of the approach keypresses (H+) and the Shannon Entropy of the avoidance keypresses (H-). Each of these variables (K+, K-, H+, H-) was produced for the five categories of facial expressions seen by subjects (producing 20 such measures). Lastly, loss aversion (LA) metrics for the keypress task included the slope of the negative curve (s-) and the slope of the positive curve (s+) from the RPT graph of the variables KH, along with absolute value of their ratio.
(XLSX)
